# Implementing a mHealth-Based Patient and Nurse Educational Program to Reduce Wound Infection in Rural Philippines

**DOI:** 10.5334/aogh.3834

**Published:** 2022-08-29

**Authors:** Valentín Henarejos, Kathleen O’Connor, Antonio Barrasa, Antonio Villalonga, Consolación Pastor, Juan Carlos Puyana, Belén Merck

**Affiliations:** 1 CEU Cardinal Herrera University Faculty of Health Sciences, Surgery, Moncada, Valenciana, ES; 2 University of Pittsburgh School of Medicine, M.D. Candidate, Pittsburgh, PA, US; 3 Santa Maria Josefa Foundation Hospital, General Surgery, Iriga City, Camarines Sur, PH; 4 University of Pittsburgh School of Medicine, Surgery, Pittsburgh, PA, US

**Keywords:** surgical wound infection, developing countries, telemedicine, patient education as topic, infection control

## Abstract

**Background:**

Surgical site infection (SSI) is a prevalent but preventable complication in low-and-middle income countries (LMIC), with reported incidence varying from 8–30%. In 2018, the MEDIPINAS surgical mission to the Philippines observed a 28.8% rate of SSI despite adherence to WHO intraoperative protocols.

The objective of this study was to introduce an educational program for wound care and early identification of wound infection. This program included provision of wound care materials and systematic protocol to ensure adequate and effective follow-up. Barriers to SSI prophylaxis in the Philippines include limited resources in regional hospitals and isolation of patients living in rural areas. The MEDIPINAS mission utilized mobile health software to connect with regional providers and to reinforce the wound care educational program introduced at discharge.

**Methods:**

The 2019 MEDIPINAS mission returned to the Philippines and operated on 187 patients in San Antonio de Padua Diocesan Hospital and Santa Maria Josefa Hospital. Before discharge, patients were individually consulted about maintaining the cleanliness of their surgical wound. Each patient was discharged with a wound care infographic and a kit to change dressings. In collaboration with regional care providers, we utilized a secure mobile health software to monitor wound healing 7 and 30 days following the operation.

**Results:**

Between the 2018 and 2019 surgical missions, we observed a decrease in SSI from 28.8% to 9.7%. Fourteen patients (7.5%) were lost to follow-up. Postoperative infection was diagnosed using photos provided by hospital-based nurses. Individuals with infections were treated with antibiotics and all but two SSI resolved after 30 days.

**Conclusions:**

Patient education, discharge with basic sanitary resources, and development of a mobile health-based follow-up infrastructure may contribute to significant reductions in SSI in LMICs. A limitation to implementation of such a program was integration of the mobile health software into the practice of local healthcare providers.

## Introduction

The Philippines is home to over 100 million people distributed over 2000 islands. While still classified as a low-to-middle-income country (LMIC) by World Bank, it has recently experienced significant economic growth without proportional improvement in health outcomes [1,2]. As a country in economic transition, the Philippines faces the “triple burden of disease,” particularly in rural areas. PhilHealth, the recently established system of universal healthcare, is strained by the existing prevalence of preventable infectious disease, new incidence of noncommunicable diseases, and other pressures from climate change and globalization [3]. Due to unequal distribution of resources, barriers to obtaining health insurance, and cultural norms regarding help-seeking behavior, elective surgical conditions are infrequently addressed [1].

The MEDIPINAS project is an international collaborative surgical initiative (ICSI) led by a group of Spanish surgeons seeking to improve access to surgical services among citizens since 2016. In February 2018, several surgeons and medical students from Cardinal Herrera CEU University traveled to the Philippines. In collaboration with a local team lead by Dr. Consolación Pastor, we carried out surgical interventions on a total of 164 patients at Santa Maria Josefa Hospital in Iriga City. Following surgery, patients were briefly instructed on postoperative wound care.

Upon follow-up one month after surgery, rates of surgical site infection (SSI) were 28.8%. This rate is surprisingly high, even given the great variability of 8–30% among published rates of postoperative SSI in LMICs [4]. Multiple studies have noted the prevalence and economic burden of SSI in LMIC, but few have instituted a cost-effective educational program to improve wound care and reduce postoperative infection [5].

To further ascertain possible causes that could explain this unanticipated high rate of infections, we looked into the environmental factors associated with the postoperative care provided to these patients. We hypothesized that improving wound care resources and introducing elemental but early monitoring techniques in collaboration with local providers would be effective in reducing SSI.

Three interventions were chosen and systematically elaborated to address to specific gaps identified as potential causes for wound infection. The first intervention was providing patient education on wound care after discharge. The second was providing access to affordable wound care cleaning materials including clean water and fresh dressings for the operation site. The third was developing a simple mobile health-based software to monitor wound healing and to reinforce the communication between the investigators and local care providers. We chose to pilot an mHealth follow-up because previous work suggests that mobile phones are highly utilized in low-resource settings [21]. Such allows for technological integration otherwise impossible without extensive infrastructure shifts.

We returned to Philippines in 2019 and offered this educational intervention to a new set of patients receiving surgical care over a three-week period in collaboration with the local team leader, Dr. Consolación Pastor. In comparing rates of SSI following our 2018 and 2019 interventions, we were able to assess the efficacy of our cost-effective educational and follow-up protocols.

## Methods

### Educational Protocol and Wound Care Intervention

Upon returning to the Philippines in 2019, we began a prospective study of our newly- implemented SSI management plan and educational protocol. The educational materials incorporated information from “Decálogo del Proyecto Infección Quirúrgica Zero,” a ten-step initiative designed in Spain to mitigate the risk of postoperative infection. To adapt to the limited resources available at our operation sites, we modified the infection-mitigating steps to emphasize intense hand washing, preparation of the surgical field with 2% alcoholic chlorhexidine, and prioritization of preoperative glycemic control for diabetic patients in accordance with WHO guildlines [6]. The study was conducted at the San Antonio de Padua Diocesan Hospital in Masbate, which contains 30 beds without surgical service, and at Santa Maria Josefa Hospital in Iriga City, which contains 100 beds and several surgical services.

This work has been approved by the Ethics Committee of the Faculty of Health Sciences at Cardenal Herrera CEU University. Compliance with the requirements for exchange of information for the purpose of diagnosis and research aligns with Royal Decree 65/2006 (January 30). Patient-identifying information was removed from medical records and all patient information was exchanged in a confidential system in compliance with Organic Law 15/1999 (December 13), with the Protection of Personal Data and Order SSI/81/2017 (January 19), and with basic guidelines to protecting patient privacy for health sciences students and professionals.

After the preoperative visit and meeting with the MEDIPINAS surgical team, patients were enrolled in the study following the model for informed consent proposed by WHO. Postoperatively, they were discharged with an infographic providing information about best practices for caring for a surgical wound with photos of SSI warning signs. Additionally, patients were discharged with a package containing saline, disinfectant, gauze, and sterile dressings. Aside from the supplies and information provided at discharge, the surgical team explained the purpose of each item in the bag and emphasized the information laid out by the infographic. Patients were encouraged to return to the hospital upon noticing signs of infection and to bring up emerging concerns at their follow-up visit. All educational information was also shared with family or support persons if present.

### mHealth-Based Monitoring Protocol

To provide surgeon oversight of the wound healing process, we introduced a simple mHealth application by utilizing a password-protected spreadsheet to connect the investigators and the Filipino nurses providing postoperative care. This exchange platform permitted the investigators and local care providers to exchange photos of healing wounds. Patient names were codified and operations were identified by the date and time of intervention, patient age, sex, comorbid conditions, anesthesia type, diagnosis to be surgically addressed, technique of surgical intervention, type of surgical wound closure, post-surgical treatment, as well as dates and comments on each uploaded photo of the surgical site.

As a baseline, we took photos of the surgical area directly before the incision and upon completion of the operation and uploaded them into the shared password-protected system. The software allowed nurses to upload photos of the wound as it healed throughout the 30-day follow-up period so that the surgical team could initiate early intervention for SSI. After 30 days, the healing process was chromatically codified: green signifying no infection throughout the healing period, yellow signifying incidence of infection at some point over the 30-day period that had since resolved, and red signifying an active SSI.

Once the follow-up period closed, we assessed the incidence of SSI in comparison to 2018 to assess the efficacy of the instruction of our surgical team, resources provided with discharge, and the thread of communication with hospital-based Filipino nurses in reducing SSI incidence.

### Public Involvement

Patients were not involved in the design of the interventions or in follow-up structure, but local care providers were involved. They gave feedback regarding the mHealth system and its integration into the care landscape. Additionally, they continue to collaborate with the MEDIPINAS team to best adapt the mHealth system to the needs of the patients they serve for future ICSIs.

## Results

Between March 19 to April 5, 2019, the MEDIPINAS19 ICSI operated in two hospitals on two islands of the Bicol Region. San Antonio de Padua Diocesan Hospital is a 30-bed hospital in Masbate that treats general medicine, pediatric, and obstetric patients. The MEDIPINAS ICSI reviewed 950 patients and operated on 19 of them at San Antonio de Padua. The capability of this facility limited number of surgical candidates and breadth of surgical techniques; there was no local doctor to facilitate postoperative follow-up and no electrical surgical equipment. We also medically treated 804 patients and surgically treated 168 patients at Santa Maria Josefa Hospital in Iriga City.

Of the 187 people who received operations, 108 (58%) were female and 79 (42%) were male. The mean age was 46.3 (range: 12–81 years). Notable comorbid factors include that 18 patients (9.6%) were hypertensive and 7 (3.7%) had non-insulin-dependent diabetes. Of the total, 153 patients (81%) were operated on under general anesthesia and 22 (19%) received regional anesthetic. All patients received a single preoperative dose (625 mg) of amoxiclav. Surgical procedures performed were umbilical hernia repair, inguinal hernia repair, lumpectomy, and tumor resection.

Wounds were closed immediately following the operation except in four cases of perianal fistula. In all cases, care was taken to achieve appropriate closure of the wound: good eversion of wound edges, plane closure, and appropriate tissue tension. Simple stitches were used to close wounds in 73 patients (39%), mattress stitches were used for 59 patients (31%), and the wounds of the remaining 51 (27%) were closed with resorbable intradermal suture. All patients were prescribed 500 mg of paracetamol and 600 mg of ibuprofen postoperatively. Before discharge, each patient received a full explanation of sanitary wound management and received the kit, which included clean dressings and an infographic summary of the wound care management plan. Patients were reminded of the wound care protocols during their postoperative follow-up visits.

Patients returned to the hospital for follow-up three to seven days following their operation and again after one month. Of those who received operations and subsequent educational materials, fourteen patients (7.5%) were lost to follow-up. A total of 18 patients (9.7%) presented with signs of surgical wound infection, such as purulent wound drainage or fluid collection, during the follow-up period. Those presenting with infection were treated with another 625 mg dose of amoxicillin/clavulanate and only two SSI remained unresolved after one- month of follow-up. These patients were instructed to follow up with local hospital staff for the next three days.

## Discussion

The 2019 MEDIPINAS ICSI utilized wound care materials, educational protocol, and mHealth-based surveillance as a low cost, high impact intervention in the prevention of surgical site infection in a low resource setting. SSI is a preventable postoperative complication, yet the cultural and economic barriers characteristic of the austere environments of rural settings in LMICs like the Philippines present significant challenges to implementing preventative measures [7]. In 2016, WHO published guidelines for surgical staff to follow to prevent postoperative infection, but we observed a high rate of SSI (28.8%) following the 2018 MEDIPINAS ICSI, despite adherence to these guidelines [6]. Minimizing postoperative infection is among the greatest challenges of surgical practice in LMICs, and patient education is underrepresented in the literature as an aspect of a multifaceted approach to infection prevention [8].

### Patient Education

Patient participation is increasingly acknowledged as an important factor in SSI prevention, but prophylactic educational programs have not been evaluated for ICSIs or in the low-resource context of countries like the Philippines [9]. Still, preliminary data is promising for the role of patient education in SSI prevention. In the UK, Bullough et al. demonstrate a 50% reduction in post-Cesarean infection with patient education pamphlets and vigilant dressing management [10]. Other studies also utilized post-discharge monitoring to assess infection risk like we did in the 2019 MEDIPINAS ICSI, but they did not use a mobile health-based follow-up protocol [11]. Thus, our protocol presents a new approach to infection management in LMICs.

The 2019 MEDIPINAS postoperative protocols helped patients and their families to practice the behaviors necessary to reduce SSI. Our pamphlets emphasize hand hygiene, wound cleaning with sterile saline, antiseptic application, and dressing changes. By providing materials such as saline and gauze along with instructions for use, we achieved a reduction in SSI from 28.8% to 9.7%. Patient education is the cornerstone of the reduction of surgical site infection, so it is critical to work with local stakeholders to provide materials and present information given the healthcare culture in a particular region.

### Wound Care Materials

The cost of the materials at discharge was 3 euros per patient. This small investment offsets the greater financial burden of SSI later, especially considering that PhilHealth is more robust in subsidizing inpatient care [1]. In rural areas of the Philippines, it is cheaper and highly common to seek care from a local healer than a physician or pharmacy [7]. Such healers often administer hot infusions of leaves or herb brews for SSI. Our group has previously observed that such local remedies often are harmful for the healing process. Instead of discouraging individuals from seeking local healers, we provided them with materials and instructions to have autonomy over their own care. This prevented possible cultural conflict and confusion between different care techniques. By providing guidelines to avoid SSI to patients and their families, we helped to reinforce social support necessary to improve post-operative outcomes [12]. Our educational materials contained visual aids and summarizing statements to maximize understandability [13].

### Mobile Health

Between 2003 and 2015, there was a nearly six-fold increase in mobile phone users in the Phillippines [14]. This exponential growth outpaces other innovations in low-resource environments and presents a unique opportunity to facilitate task sharing and provide access to specialized care [15,16]. Remote areas in LMICs typically have limited electronic medical records and desktop computer access, but leveraging mobile phone prevalence allows for technological integration without large infrastructure shifts [21]. Previous work suggests that mobile phone-based communication may present a cost-effective way to support nurses and other care providers in low-resource settings [17]. Although most published data on its efficacy pertains to monitoring of infectious disease, mHealth-based care has the potential to engage both local providers and patients alike [18,19]. The 2019 MEDIPINAS utilization of mobile phone-based communication between investigators and local providers demonstrates that it may be a viable option for supporting postoperative care in rural settings. There are many applications for mHealth follow-up beyond surgical site infection monitoring. We look forward to exploring such possibilities in future interventions in the Philippines.

Despite the promise of such software, it is not without limitations. Confidentiality and cybersecurity remain evolving obstacles across healthcare systems. Additionally, integration of mHealth infrastructure into the practice model of local providers requires time, training, and open communication [16,20]. Our mHealth system was specifically utilized to manage SSI over the 30-day study period to assess the efficacy of our educational and wound care materials, so more investigation is warranted to determine the long-term potential of integrating mHealth into global surgical care.

This study represents an early foray into the use of mHealth for postoperative follow-up in low- resource rural settings and our spreadsheet-based system is just a starting point for the integration of mobile phones in global surgery. As access to mobile phones grows exponentially, the potential for app development to deliver expertise to remote settings increases globally. That being said, there is still considerable variability in technology and broadband access across low resource settings worldwide, which presents barriers to the generalizability of mHealth-based follow-up.

Despite the universal healthcare system in the Philippines, there are still discrepancies in the electronic health record that made it difficult for us to obtain prospective demographics and comorbidity-related data. Continued development of an electronic health record will allow better analysis of the factors that might predispose patients to infection or inability to present for follow-up. Between development of mobile apps and electronic health systems, there is great potential for capacity-building through technological innovation.
